# Gastrointestinal manifestations and enzyme replacement therapy in late-onset Pompe disease: insights from a cross-sectional analysis

**DOI:** 10.1186/s13023-025-04171-8

**Published:** 2026-02-10

**Authors:** Xinting Liu, Xuejiao Li, Xinyun Yao, Gang Zhu, Xueyuan Guo, Wen He, Linyan Hu, Guang Yang

**Affiliations:** 1 Department of Pediatrics, Chinese PLA General Hospital, Beijing, China; 2 Medical School of Chinese People’s Liberation Army, Beijing, China; 3 The 14th Retired Cadre Rest Home in Haidian District, Beijing, China; 4 Department of Rehabilitation Medicine, The First Medical Center, Chinese PLA General Hospital, Beijing, China

**Keywords:** Late-onset Pompe disease, Gastrointestinal symptoms, Enzyme replacement therapy, Gastrointestinal Symptom Rating Scale

## Abstract

**Background:**

Late-onset Pompe disease (LOPD) is an autosomal-recessive disorder caused by acid α-glucosidase (GAA) deficiency, typically presenting after the first year of life and resulting in systemic glycogen accumulation. Beyond motor and respiratory manifestations, gastrointestinal (GI) symptoms have been increasingly reported. Although enzyme replacement therapy (ERT) improves skeletal and respiratory function, its impact on GI symptoms remains unclear.

**Methods:**

There were 124 LOPD patients recruited to complete the Gastrointestinal Symptom Rating Scale (GSRS), together with questionnaires on clinical manifestations and demographic data. Spearman’s correlation and multiple linear regression were used to analyze the factors associated with GI symptoms.

**Results:**

In this cross-sectional study, patients receiving standard-dose ERT (20 mg/kg, biweekly) exhibited the mildest GI symptoms compared with untreated patients and those on reduced-dose ERT (< 20 mg/kg or < biweekly). Lower family income, reduced ERT dosage, requirement for respiratory support, and tongue hypertrophy or atrophy were associated with more severe GI symptoms. With increasing ERT dose, improvements in scores of halitosis, loose stools, and the dyspepsia domain were most pronounced, and the time to onset of obvious GI symptoms was correspondingly delayed. However, longer ERT duration correlated positively with constipation severity.

**Conclusions:**

GI symptoms in LOPD are influenced by income, involvement of other organs or systems, and treatment-related factors. Standard-dose ERT significantly alleviates abdominal pain, diarrhea, and dyspepsia, but may exacerbate constipation over time. Future studies should explore novel therapeutic strategies to improve GI symptoms and nutritional status in patients with LOPD.

**Supplementary Information:**

The online version contains supplementary material available at 10.1186/s13023-025-04171-8.

## Introduction

Pompe disease (PD), also known as glycogen storage disease type II (OMIM ID: 232300), is an autosomal recessive disorder caused by the deficiency of acid α-glucosidase (GAA) [1, 2]. This can lead to abnormal accumulation of glycogen in lysosomes, resulting in multi-system damage, particularly affecting skeletal, cardiac, and smooth muscle [3]. Based on the age of onset and clinical symptoms, PD can be classified into infantile-onset Pompe disease (IOPD) and late-onset Pompe disease (LOPD) [3]. IOPD typically presents with overt clinical signs at birth or within the first few months of life. Without intervention, it leads to premature death, characterized by severe cardiac hypertrophy, generalized muscle weakness, and respiratory failure [4, 5]. LOPD is more common in adolescents and adults, primarily manifesting as progressive proximal skeletal muscle degeneration and respiratory dysfunction [6, 7]. Compared to IOPD, patients with LOPD follow a more indolent course but experience a significantly reduced quality of life.

The pathophysiology of PD is not limited to skeletal and cardiac muscles but also involves smooth muscles in the respiratory, vascular, gastrointestinal (GI), and genitourinary (GU) systems [8]. In recent years, GI symptoms in patients with PD have been increasingly reported [9–13]. Karabul et al. [10] conducted a cross-sectional survey involving 57 adult PD patients and 57 controls, revealing significantly higher incidence rates of stool urgency and diarrhea compared to the control group. Bernstein et al. [11] reported three LOPD patients with various GI symptoms, such as chronic diarrhea, postprandial bloating, and abdominal pain. In patients with LOPD, severe GI symptoms can cause discomfort in patients, leading to inadequate nutritional intake, affecting their growth, development, and recovery, and further exacerbating the patient’s condition.

Recombinant human acid alpha-glucosidase (rhGAA, alglucosidase alfa) enzyme replacement therapy (ERT) is the primary treatment for PD and can improve patients’ clinical symptoms and prognosis to some extent [14]. ERT has been demonstrated to have substantial therapeutic benefits in enhancing skeletal muscle function and extending survival. However, only a few case reports existed regarding its impact on GI function [11, 15, 16]. Some studies suggested that ERT may indirectly improve swallowing function by alleviating tongue muscle weakness and enhance GI motility function by clearing glycogen accumulation in the smooth muscle of the GI tract [15, 17, 18]. Additionally, the dosage and duration of ERT, along with the high cost, may potentially influence the management of GI symptoms in patients with PD.

In this cross-sectional study, 124 patients with LOPD were enrolled to systematically characterize the severity of GI symptoms and to delineate their associations with ERT dosage, socioeconomic determinants, and other clinical variables, with the ultimate goal of generating evidence-based therapeutic recommendations to improve nutritional status and quality of life.

## Materials and methods

### Study design and population

We recruited 135 patients clinically diagnosed with LOPD for this cross-sectional study. After signing the informed consent form, they were required to complete a questionnaire regarding demographic information, GI symptoms within the most recent month, and other systemic clinical manifestations, as well as provide clinical diagnostic reports. We received 124 completed questionnaires that were used for the analyses in our current study. The study was approved by the Ethics Committee of the First Medical Center of the People’s Liberation Army General Hospital.

### Patient-reported information

The questionnaire administered in this study consisted of two sections, patient-reported items and clinician-recorded variables, and required approximately 30 min to complete. Patients independently supplied demographic and non-clinical information: gender, age, body mass index (BMI, kg/m2), residential area, ethnicity, family history, educational background, occupation, and family income. They also self-reported their use of assistive devices, including mobility-assisted devices (e.g., wheelchairs, traction devices, gear shifters, and standing frames), respiratory support (e.g., suction machine, tracheotomy, and invasive/non-invasive ventilator), and nutritional support (e.g., oral/nasal feeding, gastrostomy, and intravenous nutritional support). Patients were also asked to self-assess the effectiveness of ERT based on the degree of improvement after treatment in muscle strength, left ventricular thickness on cardiac ultrasound, lung function, and creatine kinase levels. Responses were categorized into three levels: improved, unchanged, or poor efficacy.

### Medical information and medical records

The remaining data were derived from objective examinations and standardized clinical evaluations completed by the clinicians, including supplementary examination reports provided by the patients (left-ventricular thickness on echocardiography, pulmonary-function tests, serum creatine-kinase levels, and spinal X-ray or MRI findings). A diagnosis of scoliosis requires a clinical evaluation report from a professional physician or an X-ray examination of the spine [19]. Motor ability was evaluated using the World Health Organization (WHO) [20] gross motor development milestones, which were divided into six levels: independent sitting, dependent standing, crawling, dependent walking, independent standing and independent walking. Each acquired motor milestone was scored as one point, with a total score of six points. Tongue hypertrophy or atrophy represents manifestations of tongue muscle involvement. Patients may exhibit corresponding signs on MRI or clinical presentations, such as the tongue being unable to be fully accommodated within the oral cavity, exposure of the tongue tip or lateral borders, tongue shortening, or difficulty in tongue protrusion [21, 22]. Disease-specific clinical variables comprised age at diagnosis and onset of LOPD, duration, dose and efficacy of ERT, disease duration, GSRS scores, and other clinical manifestations.

### Gastrointestinal symptom rating scale (GSRS)

The GSRS questionnaire is a self-inquiry scale first developed by Svedlund in 1988 and subsequently validated for clinical use [23]. It comprises 15 items, each scored on a 4-point Likert scale ranging from 0 to 3, representing no symptoms, mild symptoms, moderate symptoms, and severe symptoms, respectively. The total score of the symptoms ranged from 0 to 32 points. Patients scoring ≥ 16 were categorized as having obvious GI symptoms, indicating that at least one item received a score ≥ 2. Assign similar symptoms from 15 items to five domains: abdominal pain (abdominal pain, hunger pain, and nausea), reflux syndrome (heartburn and acid regurgitation), diarrhea syndrome (diarrhea, loose stools, and urgent need for defecation), indigestion syndrome (borborygmus, bloating, burping, and halitosis), and constipation syndrome (constipation, hard stools, and a feeling of incomplete evacuation) [23–25]. Patients should fill out depending on the level of GI symptoms over the previous month.

### Enzyme replacement therapy (ERT)

Patients who were receiving intravenous infusions of recombinant human acid alpha-glucosidase (rhGAA) with 20 mg/kg every two weeks were considered to have received standard treatment [26, 27]. Infusion dosages below 20 mg/kg or administration frequencies of less than biweekly were categorized as diminished amounts of ERT. We defined the period of not receiving ERT as the absence of intravenous rhGAA infusions within the past year.

### Statistical analysis

Statistical analysis was performed using IBM SPSS version 27.0 software (IBM Corporation, Armonk, NY, USA). The Kolmogorov-Smirnov test was used for evaluating the normality of demographic and clinical data. Continuous variables were reported as averages ± standard deviations or median (interquartile range) according to the normality. Categorical data were expressed as frequencies (%). Compare the differences between GSRS scores and other measurements using independent samples t-test or analysis of variance (ANOVA) for normally distributed continuous data and a Mann–Whitney U test or Kruskal–Wallis test for non-normally distributed continuous data. Categorical data were analyzed using the chi-squared test. The correlations among GSRS scores and age/age of diagnosis/age of onset (continuous), sex (category, male/female), BMI (continuous), disease duration (continuous), residential area (category, northern regions of China = 1/southern regions of China = 2), occupation (category, work or study = 1/no work = 2), family income (continuous), duration of ERT (continuous), dose of ERT (category, without ERT = 1/reduced-dose ERT = 2/standard-dose ERT = 3), efficacy of ERT (category, poor = 1/unchanged = 2/improved = 3), need respiratory support (category, yes = 1/no = 2), need mobility assisted devices (category, yes = 1/no = 2), need nutritional support (category, yes = 1/no = 2), scoliosis (category, yes = 1/no = 2), developmental milestones (continuous), and tongue hypertrophy or atrophy (category, yes = 1/no = 2) were analyzed with the Spearman r correlation coefficient. Multiple linear regression analyses were used to explore associations between GSRS scores and the results of bivariate analysis. For all statistical analyses, the *p* value ≤ 0.05 was considered statistically significant. Kaplan Meier curves were used to evaluate the time differences in obvious GI symptoms among patients treated with different doses of ERT, with statistical significance evaluated by the log-rank test.

## Results

### Characteristics of the patients

In our study, 124 patients were included: 57 males (45.97%) and 67 females (54.03%), with an average age of 22.73 ± 12.56 years (Table 1). Thirty-four individuals were receiving adequate and regular ERT treatment, forty-one patients were receiving treatment with extended intervals or reduced doses, and forty-nine people were currently not receiving ERT treatment. For patients receiving ERT, the mean age at initiation was 21.79 ± 12.18 years, and the average duration of treatment was 1.99 ± 1.83 years (Table 2).

**Table 1:** Demographic characteristics of the patients with different doses of ERT

Characteristics	Without ERT	Reduced-dose ERT	Standard-dose ERT	*p* value
Patients (n)	49	41	34	
Gender				0.432
Male, n (%)	27 (55.10)	18 (43.90)	12 (35.29)	
Female, n (%)	22 (44.90)	23 (56.10)	22 (64.71)	
Age, Mean ± SD, (years)	22.35 ± 12.59	25.51 ± 11.47	21.68 ± 12.46	0.291
BMI, Mean ± SD, (kg/m2)	16.96 ± 4.36	18.44 ± 3.99	16.13 ± 3.21	0.029*
Residential area				0.416
Northern regions of China, n (%)	22 (44.90)	25 (60.98)	14 (41.18)	
Southern regions of China, n (%)	27 (55.10)	16 (39.02)	20 (58.82)	
Occupation				0.384
Work or study	23 (46.94)	25 (60.98)	20 (58.82)	
No work	26 (53.06)	16 (39.02)	14 (41.18)	
Family income, Mean ± SD, (Thousand Yuan/year, RMB)	72.57 ± 7.65	73.05 ± 5.57	90.59 ± 6.27	0.141

Abbreviations: n, number of patients; SD, standard deviation; BMI, body mass index; ERT, enzyme replacement therapy

**Table 2:** Clinical characteristics of the patients with different doses of ERT

Characteristics	Without ERT	Reduced-dose ERT	Standard-dose ERT	*p* value
Age at diagnosis, Mean ± SD, (years)	16.24 ± 10.02	19.70 ± 11.18	15.21 ± 9.75	0.180
Age at onset, Mean ± SD, (years)	11.68 ± 9.08	14.27 ± 8.04	11.43 ± 7.94	0.146
Duration of ERT, Mean ± SD, (years)	NA	1.96 ± 2.16	2.02 ± 1.35	0.347
Disease duration	10.67 ± 6.65	11.24 ± 8.41	10.24 ± 7.74	0.840
GSRS scores	9.37 ± 8.71	7.05 ± 6.71	4.71 ± 4.69	0.044*
Efficacy of ERT, n (%)				0.114
Poor	NA	6 (14.63)	5 (14.71)	
Unchanged	NA	21 (51.22)	12 (35.29)	
Improved	NA	14 (34.14)	17 (50.00)	
Need respiratory support, n (%)	28 (57.14)	24 (58.54)	19 (55.88)	0.357
Need mobility assisted devices, n (%)	17 (34.69)	11 (26.83)	10 (29.41)	0.817
Need nutritional support, n (%)	5 (10.20)	5 (12.20)	4 (11.76)	0.959
Scoliosis, n (%)	25 (51.02)	21 (51.22)	13 (38.24)	0.225
Developmental milestone score, Mean ± SD	4.31 ± 2.24	5.05 ± 1.82	5.29 ± 1.62	0.044*
Tongue hypertrophy or atrophy, n (%)	12 (24.49)	5 (12.20)	7 (20.59)	0.921

Abbreviations: SD, standard deviation; ERT, enzyme replacement therapy; LOPD, late-onset Pompe disease; GSRS, gastrointestinal symptom rating scale; n, number of patients

Overall, patients who received ERT had milder GI symptoms and achieved more motor milestones (5.99 ± 5.96/5.16 ± 1.72) when compared to patients without ERT (9.37 ± 8.71/4.31 ± 2.24) (*p* = 0.038, *p* = 0.020). Patients who have received regular treatment (4.71 ± 4.69/5.29 ± 1.62) were more likely to present with milder clinical symptoms compared to those receiving reduced doses (7.05 ± 6.71/5.05 ± 1.82) (*p* = 0.044, *p* = 0.044). However, there were no significant differences among the three groups in terms of age, gender, disease duration, use of mobility aids, nutritional or respiratory support, or self-reported treatment efficacy (*P* > 0.05). Among patients receiving full-dose ERT, half of them (17/34, 50.00%) reported significant clinical improvement. Ten patients (10/34, 29.41%) indicated that their motor function, such as muscle strength, had improved. Four individuals (4/34, 11.76%) self-reported alleviation of GI symptoms along with weight gain. Four patients (4/34, 11.76%) experienced improvements in respiratory function. Additionally, liver function markers—including aspartate aminotransferase (AST), alanine aminotransferase (ALT), and total cholesterol—were normalized in one patient (1/34, 2.94%). In contrast, among those receiving reduced-dose ERT, 14 patients (14/41, 34.14%) reported notable improvement following the treatment. Of these, nine patients (14/41, 21.95%) experienced better motor function, such as improved gait stability and stair-climbing ability. Five individuals (5/41, 12.20%) benefited from enhanced respiratory function, and one patient (1/41, 2.44%) showed concurrent improvement in gastrointestinal.

### Factors associated with GI symptoms

Spearman’s correlation analysis showed that GSRS scores correlated with family income (*r* = -0.211, *p* = 0.019), need respiratory support (*r* = -0.254, *p* = 0.004), scoliosis (*r* = -0.211, *p* = 0.019), tongue hypertrophy or atrophy (*r* = -0.264, *p* = 0.003), and dose of ERT (*r* = -0.223, *p* = 0.013). Patients with lower family income and dosages of ERT, as well as those who required respiratory support and had tongue hypertrophy or atrophy, experienced more severe GI symptoms. In contrast, the GSRS scores were positively associated with age (*r* = 0.244, *p* = 0.006), disease duration (*r* = 0.269, *p* = 0.003), and age of diagnosis (*r* = 0.215, *p* = 0.017) (Table 3). According to the multiple linear regression analysis, the interaction between changes in scoliosis, family income, and tongue hypertrophy or atrophy contributed to explaining changes in GSRS scores after adjustments for covariates (R^2^ = 0.253, Adj. R^2^ = 0.215, F = 6.609, *p* < 0.001. Table 4).

**Table 3:** Results of spearman’s correlation analysis between GSRS scores and other measurements

Other measurements	*r* value	*p* value
Age	0.244	0.006**
Gender	-0.130	0.151
BMI	0.139	0.124
Residential area	0.064	0.477
Occupation	0.138	0.126
Family income	-0.211	0.019*
Disease duration	0.269	0.003**
Age at diagnosis	0.215	0.017**
Age at onset	0.150	0.096
Duration of ERT	0.179	0.123
Dose of ERT	-0.223	0.013*
Efficacy of ERT	-0.049	0.675
Need respiratory support	-0.254	0.004**
Need mobility assisted devices	-0.096	0.289
Need nutritional support	-0.021	0.816
Scoliosis	-0.211	0.019*
Developmental milestone score	-0.144	0.111
Tongue hypertrophy or atrophy	-0.264	0.003**

Abbreviations: BMI, body mass index; LOPD, late-onset Pompe disease; ERT, enzyme replacement therapy

**Table 4:** Multiple linear regression analysis of GSRS scores with other measurements

Independent variable	Beta	SE	95% CI	*p* value
Constant term		3.937	7.604, 23.200	< 0.001***
Scoliosis	-1.719	1.231	-4.156, 0.718	0.165
Family income	-0.134	0.093	-0.319, -0.051	0.154
Dose of ERT	-1.961	0.729	-3.404, -0.518	0.008**
Tongue hypertrophy or atrophy	-4.628	1.526	-7.650, -1.607	0.003**
Need respiratory support	-1.347	1.354	-4.027, 1.334	0.322
Disease duration	0.159	0.085	-0.009, 0.326	0.064

Abbreviations: SE, standard error; Beta, standardized regression coefficient; ERT, enzyme replacement therapy; LOPD, late-onset Pompe disease

In the models of the five GSRS domains, dose of ERT and tongue hypertrophy or atrophy were slightly associated with abdominal pain (β=-0.189, *p* = 0.031; β=-0.191, *p* = 0.032), indigestion (β=-0.237, *p* = 0.006; β=-0.189, *p* = 0.026), and diarrhea risk (β=-0.235, *p* = 0.005; β=-0.294, *p* < 0.001); the scoliosis and tongue hypertrophy or atrophy were associated with the scores of reflux syndrome (β=-0.214, *p* = 0.016; β=-0.237, *p* = 0.006). Unexpectedly, the duration of ERT was positively associated with the severity of constipation (β = 0.253, *p* = 0.037).

### Severity of GI symptoms at different ERT doses

**Fig. 1:**
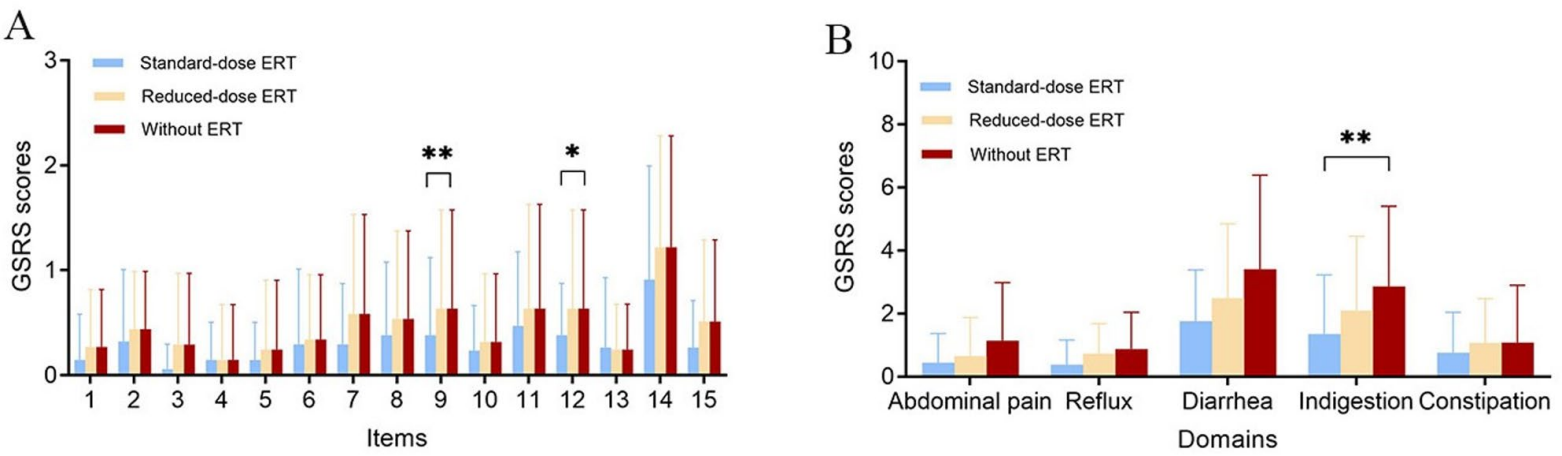
The relationship between ERT dosage and items in GSRS scores. (**A**) The relationship between the scores of 15 items in GSRS and the dose of ERT. The horizontal axis 1–15 respectively designate: abdominal pain, heartburn, acid regurgitation, hunger pain, nausea, borborygmus, bloating, burping, halitosis, constipation, diarrhea, loose stools, hard stools, urgent need for defecation, and feeling of incomplete evacuation. (**B**) The correlation between the results of 5 domains in GSRS and the dosage of ERT. The horizontal axis 1–5 respectively represent: abdominal pain, reflux syndrome, diarrhea syndrome, indigestion syndrome, and constipation syndrome. Values of GSRS were expressed as mean ± SD, * represented *p* < 0.05, ** represented *p* < 0.01

Figure 1 illustrates the effects of different ERT dosages on GI function using the Kruskal–Wallis H test. Significant differences were observed in the improvement of halitosis and loose stool scores with increasing ERT dosage (*p* = 0.009 and *p* = 0.026, respectively). Among the three ERT groups, patients without ERT had the highest mean GSRS scores for halitosis and loose stools (0.96 ± 1.00 and 0.96 ± 1.00, respectively). Patients receiving the standard ERT dose showed the mildest gastrointestinal symptoms (0.38 ± 0.74 and 0.38 ± 0.49), while those on a reduced-dose had intermediate scores (0.63 ± 0.94 and 0.63 ± 0.94).

In addition, among the five GSRS domains, the indigestion syndrome showed the most pronounced improvement trend with increasing ERT dosage (*p* = 0.008). The mean indigestion scores were 2.86 ± 2.55 in the ERT-naïve group, 2.10 ± 2.35 in the reduced-dose group, and 1.35 ± 1.87 in the standard-dose group.

**Fig. 2:**
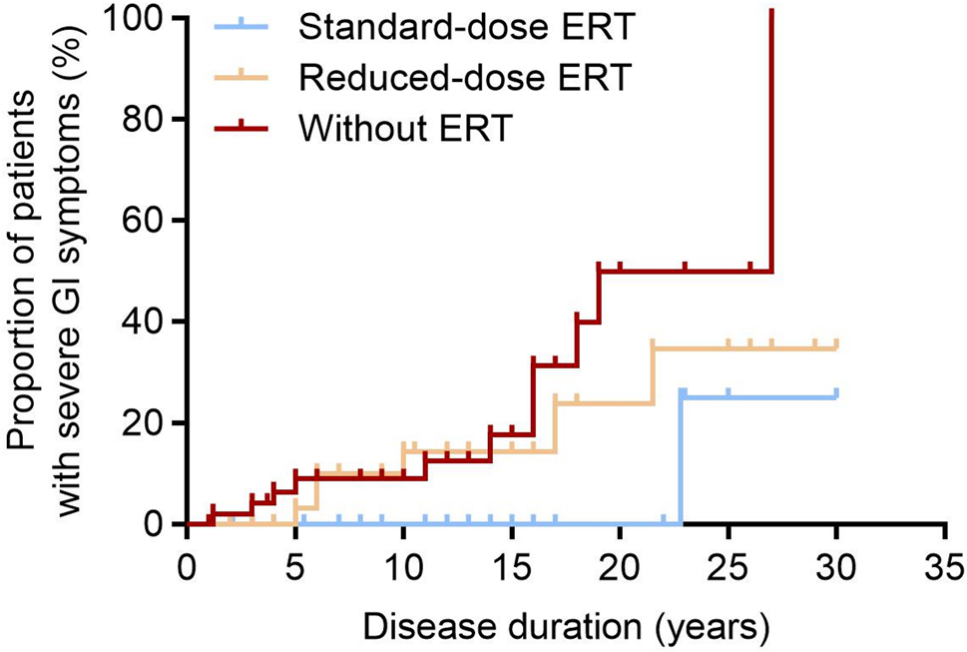
Kaplan-Meier curves depict the outcomes for disease duration and severe GI symptoms in Pompe patients stratified by ERT-dose group. The log-rank test revealed a significant difference in the time to onset of severe GI symptoms among the three groups (without ERT, reduced-dose ERT, and standard-dose ERT) during disease progression (*p* < 0.05)

Kaplan–Meier analysis revealed that the duration free of obvious GI symptoms was significantly shorter in patients receiving reduced-dose or no ERT than in those on standard-dose ERT (log-rank *p* = 0.014) (Fig. 2).

## Discussion

In this study, we found that patients receiving a standardized dose of ERT exhibited significantly milder GI symptoms compared to those who were untreated or on a reduced-dose regimen. Lower family income, reduced ERT dosage, requirement for respiratory support, and the presence of tongue hypertrophy or atrophy were independently associated with more severe GI symptoms. Notably, halitosis, loose stools, and dyspepsia showed the greatest improvement as ERT dose increased. These findings have highlighted the importance of ERT not only in managing motor and respiratory symptoms but also in addressing GI complications in LOPD [9, 11, 13].

The investigation revealed that patients with lower family income tended to experience more severe GI symptoms (*r* = -0.211). Limited family income may restrict patients’ ability to adhere to ERT or to access additional supportive treatments such as nutritional support and rehabilitation training. Moreover, low income can contribute to psychological stress, which may exacerbate GI symptoms [28–30]. Patients who had never received ERT and those presenting with scoliosis, tongue hypertrophy or atrophy, or requiring respiratory support exhibited significantly more severe gastrointestinal manifestations. Scoliosis may alter the anatomical position and spatial structure of the esophagus, stomach, and intestine, thereby disrupting the normal peristalsis and emptying patterns of the GI tract, and bring digestive system symptoms to patients, especially heartburn and acid regurgitation [31, 32]. Tongue hypertrophy or atrophy impairs chewing and swallowing, preventing adequate initial digestion of food within the oral cavity [33, 34]. Patients requiring respiratory support also exhibited more severe GI symptoms. Mechanical ventilation increases intrathoracic pressure, predisposing to reflux syndrome. Previous studies have demonstrated that high positive end-expiratory pressure (PEEP) ventilation reduces splanchnic blood perfusion, potentially compromising the energy supply to gastrointestinal smooth muscle and impairing contractility, thereby contributing to symptoms such as dyspepsia [35].

In comparison with both a previously published study for healthy adults and our survey of healthy children, GSRS scores were markedly higher in patients with LOPD [36] (Supplementary Fig. 1). Importantly, the young age profile of this cohort (23.21 ± 12.20 years) allowed us to focus on “early-stage” GI that emerge early in the natural history of LOPD, before the confounding impact of long-term complications such as profound muscle weakness. The presence of overt GI dysfunction at this initial disease phase indicates that gastrointestinal involvement may constitute an early systemic feature of LOPD. In addition, different doses of ERT had varying effects on GI symptoms. As the ERT dosage increased, the degree of improvement in symptoms such as halitosis and loose stools became significantly more pronounced. Among the five domains of the GSRS, the indigestion syndrome showed the most prominent trend of improvement. It indicates that ERT can effectively restore GI function in LOPD, particularly for functional dyspepsia-related symptoms, and that early intervention before GI complaints become chronic may effectively slow their evolution.

Nevertheless, prolonged or high-dose ERT necessitates continuous monitoring of gastrointestinal function. We observed that the duration of ERT was positively correlated with the severity of constipation. We speculate that this may be due to potential adverse effects of long-term ERT, possibly altering the gut microbiota or intestinal function, although this hypothesis has not yet been confirmed by clinical studies. Therefore, in clinical practice, it is important to monitor changes in GI symptoms during ERT closely. If patients developed severe constipation or new GI symptoms that were not present prior to treatment, temporary discontinuation of ERT or adjunctive therapy with prokinetic agents should be considered. Previous studies have reported that diarrhea, abdominal pain, and reflux were the most common GI symptoms in LOPD patients [9, 11, 16, 37, 38]. However, constipation has also been documented. Gastrointestinal symptoms were also frequently observed in several lysosomal storage disorders. Banikazemi et al. [39] reported three patients with Fabry disease who suffered from postprandial abdominal pain, bloating, and severe diarrhea. Zimran et al. [40] described patients with Gaucher disease with weight loss and abdominal pain, and their GI symptoms were resolved through ERT [41]. The accumulation of glycogen in the tongue, esophagus, gastrointestinal smooth muscles, and spinal motor neurons may be the pathophysiological mechanism underlying the GI symptoms in patients with LOPD. In the Gaa^−/−^ mouse model, there was widespread glycogen accumulation in the smooth muscle cells of the aorta, trachea, esophagus, stomach, and bladder [42]. In addition, there was an increased abundance of both lysosome membrane protein (LAMP1) and autophagosome membrane protein (LC3), which may lead to pathological changes in smooth muscle cells [9, 43]. Autopsy findings from patients with LOPD revealed glycogen accumulation and vacuoles in the muscles of the tongue, esophagus, and small intestine [17, 44]. Substantial glycogen accumulation was also observed in the spinal cord and motor neurons [45]. The accumulation of glycogen in spinal motor neurons innervating the digestive tract may lead to dysfunction in sphincter contraction and digestive gland secretion, particularly at the lower esophageal sphincter (LES) and the internal anal sphincter (IAS), which are intimately linked to reflux and constipation. Consequently, these pathological changes could underlie the corresponding gastrointestinal symptoms observed in affected patients.

This study has several limitations. Its cross-sectional design precludes causal inferences, and reliance on self-reported questionnaires may introduce recall bias or inter-individual variability in symptom perception. The cohort is dominated by young patients with a relatively short median ERT exposure. While this feature allowed us to characterize early-stage GI manifestations, such as mild ingestion that might otherwise be overlooked, it limits conclusions regarding long-term disease progression and the durability of ERT benefits. Additionally, objective measures (endoscopic biopsies, electrogastrography, etc.) were unavailable, which potentially obscures subclinical involvement or underlying pathophysiology. Future prospective, longitudinal studies spanning multiple age groups and incorporating healthy controls, objective biomarkers, and genomic datasets are necessary to provide sufficient evidence to describe the impact of LOPD on the gastrointestinal tract, establish genotype-phenotype correlations, and improve early treatment strategies. Gene therapy has emerged as a potential successor to ERT in early-stage clinical applications. We anticipate that it will not only further ameliorate GI symptoms but also mitigate the adverse effects associated with long-term ERT administration.

## Conclusion

It was concluded that the GI symptoms in LOPD patients are associated with multiple factors, including family income, the need for respiratory support, scoliosis, tongue hypertrophy or atrophy, and ERT dosage. Standard ERT has a significant effect on improving symptoms such as diarrhea, abdominal pain, and dyspepsia. However, long-term ERT might conversely lead to constipation. In the future, gene therapy, personalized treatment, and other approaches will have the potential to improve the GI symptoms and nutritional status of LOPD patients.

## Supplementary Information

Below is the link to the electronic supplementary material.

Supplementary Material 1: Figure 1. The difference in GSRS scores between healthy controls and the LOPD group. The horizontal axis respectively represents: healthy adult controls, healthy pediatric controls, total LOPD patients in this study, LOPD patients without ERT, LOPD patients with reduced-dose ERT, LOPD patients with standard-dose ERT. Values of GSRS were expressed as mean ± SD

## Data Availability

The dataset supporting the conclusions of this article is included within the article.

## Abbreviations

ALT: Alanine aminotransferase (ALT)
AST: Aspartate aminotransferase
BMI: Body mass index
ERT: Enzyme replacement therapy
GAA: Acid α-glucosidase
GI: Gastrointestinal
GSRS: Gastrointestinal Symptom Rating Scale
GU: Genitourinary
IOPD: Infantile-onset Pompe disease
LAMP1: Lysosome membrane protein
LOPD: Late-onset Pompe disease
rhGAA: Recombinant human acid alpha-glucosidase
PD: Pompe disease
PEEP: Positive end-expiratory pressure
