# Supplementary figures and images for: Gastrointestinal manifestations and enzyme replacement therapy in late-onset Pompe disease: insights from a cross-sectional analysis
Source: Orphanet J Rare Dis. 2026 Feb 10;21:50. doi: 10.1186/s13023-025-04171-8 (PMC12892710; doi:10.1186/s13023-025-04171-8)

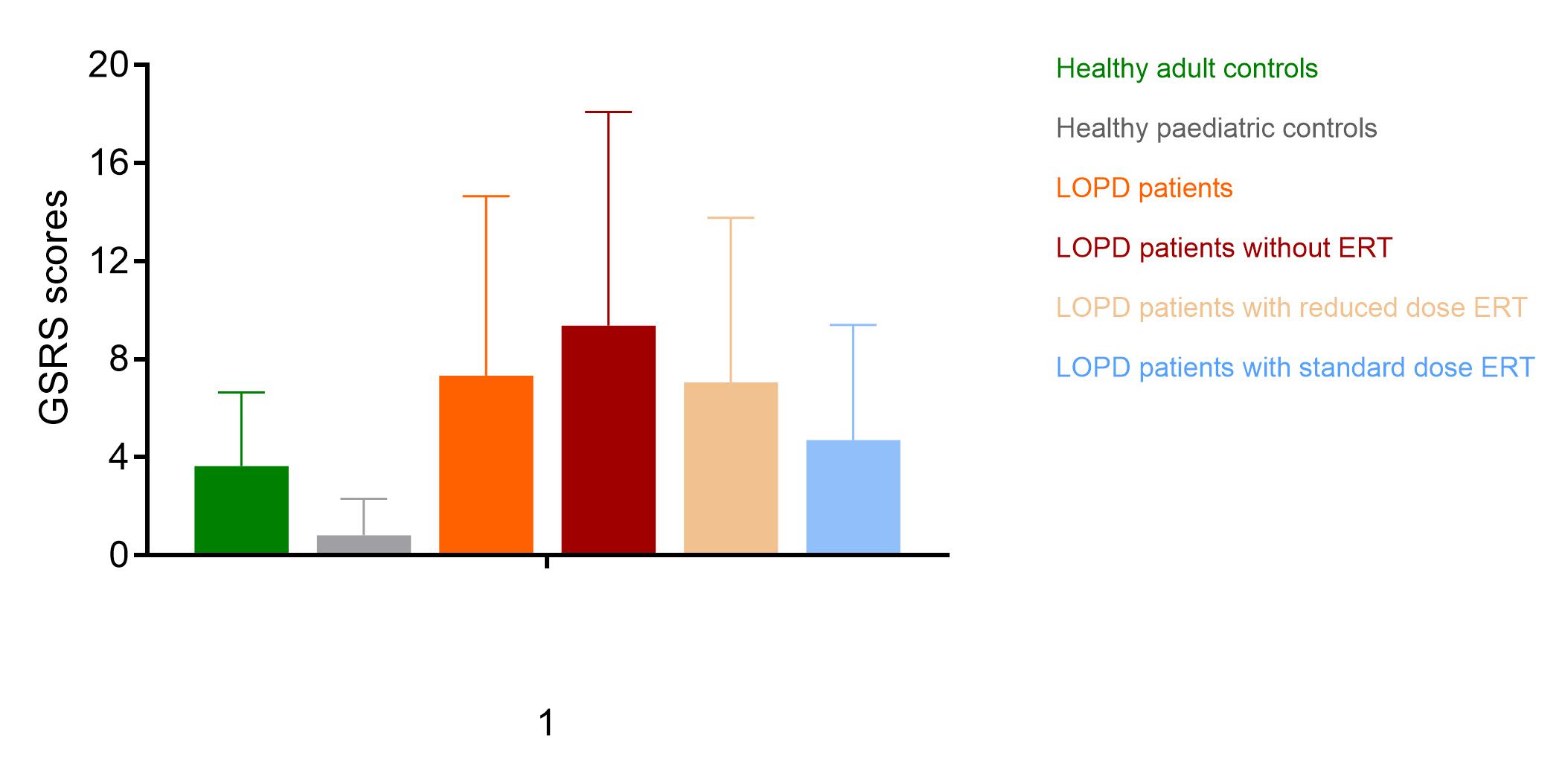

Supplement: Supplementary file 1 — Supplementary Material 1: Figure 1. The difference in GSRS scores between healthy controls and the LOPD group. The horizontal axis respectively represents: healthy adult controls, healthy pediatric controls, total LOPD patients in this study, LOPD patients without ERT, LOPD patients with reduced-dose ERT, LOPD patients with standard-dose ERT. Values of GSRS were expressed as mean ± SD [file 13023_2025_4171_MOESM1_ESM.jpg]
